# Laboratory diagnosis of emerging human coronavirus infections – the state of the art

**DOI:** 10.1080/22221751.2020.1745095

**Published:** 2020-03-30

**Authors:** Michael J. Loeffelholz, Yi-Wei Tang

**Affiliations:** a Cepheid, Sunnyvale, CA, USA; b Cepheid, Danaher Diagnostic Platform, Shanghai, People’s Republic of China

**Keywords:** Human coronavirus, SARS-CoV-2, COVID-19, POCT, real-time PCR, serology

## Abstract

The three unprecedented outbreaks of emerging human coronavirus (HCoV) infections at the beginning of the twenty-first century have highlighted the necessity for readily available, accurate and fast diagnostic testing methods. The laboratory diagnostic methods for human coronavirus infections have evolved substantially, with the development of novel assays as well as the availability of updated tests for emerging ones. Newer laboratory methods are fast, highly sensitive and specific, and are gradually replacing the conventional gold standards. This presentation reviews the current laboratory methods available for testing coronaviruses by focusing on the coronavirus disease 2019 (COVID-19) outbreak going on in Wuhan. Viral pneumonias typically do not result in the production of purulent sputum. Thus, a nasopharyngeal swab is usually the collection method used to obtain a specimen for testing. Nasopharyngeal specimens may miss some infections; a deeper specimen may need to be obtained by bronchoscopy. Alternatively, repeated testing can be used because over time, the likelihood of the SARS-CoV-2 being present in the nasopharynx increases. Several integrated, random-access, point-of-care molecular devices are currently under development for fast and accurate diagnosis of SARS-CoV-2 infections. These assays are simple, fast and safe and can be used in the local hospitals and clinics bearing the burden of identifying and treating patients.

## Agent

Coronaviruses belong to the family *Coronaviridae* which includes four genera, *Alphacoronavirus*, *Betacoronavirus*, *Deltacoronavirus* and *Gammacoronavirus*, as well as several subgenera and species. Coronaviruses are found in a variety of animals and humans. Human coronaviruses (HCoVs) include HCoV-229E and HCoV-NL63 in the genus *Alphacoronavirus*, and HCoV-OC43 and HCoV-HKU1 in the A lineage (subgenus *Embecovirus*) of genus *Betacoronavirus*. HCoVs were first isolated in cell culture in the 1960s from persons with upper respiratory infections. These were later characterized as HCoV-229E and HCoV-OC43 [1]. HCoV-NL63 and HCoV-HKU1 were discovered in the early 2000s from persons with bronchiolitis and pneumonia. In 2002 a *Betacoronavirus* in lineage B (subgenus *Sarbecovirus*) originating in bats, then spread from civets to humans in the Guangdong province of southern China, causing severe respiratory disease, and taking the name severe acute respiratory syndrome-related coronavirus (SARS-CoV) [2,3]. In 2012 a *Betacoronavirus* in lineage C (subgenus *Merbecovirus*) spread from camels to humans in Saudi Arabia, causing a similar clinical syndrome as SARS, taking the name Middle East respiratory syndrome-related coronavirus (MERS-CoV) [4–6].

The latest coronavirus to emerge in humans appeared in Wuhan City, Hubei Province, China in December 2019 [7,8] and has been designated SARS-CoV-2 [9]. Genomic sequencing shows SARS-CoV-2 to be closely related to betacoronaviruses detected in bats (88% sequence identity), but distinct from SARS-CoV (79% sequence identify) [8,10]. SARS-CoV-2 is taxonomically related to the subgenus *Sarbecovirus* together with SARS-CoV and bat SARS-like CoVs [11]. Phylogenomic evaluations of coronaviruses circulating in China can be view in http://diverge.hunter.cuny.edu/~weigang/mobile-cov/?from=singlemessage&isappinstalled=0 (Accessed 1 March 2020).

Coronaviruses are enveloped viruses containing a single strand of positive-sense RNA. Virions are mostly spherical, with pronounced spiked glycoprotein (S) embedded in the envelope. Additional structural proteins include envelope (E), matrix (M), and nucleocapsid (N). Intra- and inter-species transmission of CoVs, and genetic recombination events contribute to the emergence of new CoV strains [1].

## Clinical and public health significance

### Epidemiology

HCoVs are endemic (HCoV-229E, HCoV-NL63, HCoV-OC43 and HCoV-HKU1) or epidemic (SARS-CoV, MERS-CoV and SARS-CoV-2). In temperate regions endemic HCoVs usually display a winter seasonality, although HCoV-229E has been detected sporadically throughout the year [12]. Endemic HCoVs are globally distributed and are maintained in the human population. The SARS-CoV pandemic came to an end in 2003 (https://www.who.int/csr/resources/publications/CDS_CSR_ARO_2004_2.pdf?ua=1. Accessed 3 February 2020), less than a year after the first reported case. In contrast, human cases caused by MERS-CoV continue to be reported at the time of writing, more than seven years after the first reported case. Most laboratory-confirmed MERS cases have occurred in the Eastern Mediterranean Region, and the majority of those in Saudi Arabia. Unlike the endemic HCoVs, SARS-CoV and MERS-CoV are maintained in zoonotic reservoirs. The SARS and MERS outbreaks were driven in part by super-spreading events in which individuals directly infected a disproportionally large number of contacts [13]. The SARS-CoV-2-caused coronavirus disease 2019 (COVID-19) epidemic originated in a Wuhan, China market that sold exotic animals for consumption. Based on genetic relatedness to other betacoronaviruses, SARS-CoV-2 likely has a zoonotic reservoir. However, the precise source of SARS-CoV-2 that initially infected humans remains to be confirmed. The SARS-CoV-2 appears to be substantially more contagious than SARS-CoV (Table 1). The distribution of SARS-CoV-2 in different mammalian species is unknown. An interesting question is the susceptibility of farm animals and pets, and their role in the epidemiologic cycle as their angiotensin-converting enzyme 2 (ACE2) receptor shares similarity with human ACE2 [14].

**Table 1.** Human coronaviruses.

Virus	Genus	Disease	Discovered	References
CoV-229E	Alpha	Mild respiratory tract infection	1967	[31]
CoV-NL-63	Alpha	Mild respiratory tract infection	1965	[32]
CoV-HKU-1	Beta	Mild respiratory tract infection; pneumonia	2005	[54]
CoV-OC43	Beta	Mild respiratory tract infection	2004	[68]
SARS-CoV	Beta	Human severe acute respiratory syndrome, 10% mortality rate	2003	[2,3]
MERS-CoV	Beta	Human severe acute respiratory syndrome, 37% mortality rate	2012	[4–6]
SARS-CoV-2	Beta	Severe acute respiratory infections, <2% mortality rate	2019	[8,20,22,59]

### Symptoms

Infections caused by endemic HCoVs have an incubation period of 2–5 days and are associated with mild upper respiratory symptoms (the “common cold”). Endemic HCoVs are among the most frequent cause of upper respiratory tract infections. Lower respiratory tract infections (bronchiolitis, pneumonia) are rare. Following an incubation period of usually 4–5 days, patients infected with SARS-CoV often present with symptoms of fever, headache, and myalgias. Respiratory symptoms including cough and dyspnoea usually develop from several days to a week after illness onset. Atypical pneumonia and respiratory deterioration occur in 20–30% of cases. The incubation period and clinical course of MERS are similar to that of SARS, the exception being a higher proportion of cases progressing to respiratory deterioration and distress. The incubation period and clinical course of SARS-CoV-2 infection are probably similar to that of SARS. Li et al. first reported a mean incubation period of 5.2 days [15]. Fever and cough are frequently reported early in the course of illness [16,17]. Infections are also characterized by dyspnoea, respiratory distress and positive chest X-ray [10]. Lower respiratory symptoms often develop about 1 week from the onset of initial symptoms [16].

### Morbidity and mortality

Globally over 8000 cases and over 900 deaths due to SARS-CoV were reported, with a case-fatality ratio of approximately 11% (https://www.who.int/csr/sars/en/WHOconsensus.pdf. Accessed 3 February 2020). Between September 2012 and November 2019, there were 2494 laboratory-confirmed cases of MERS, with 858 deaths (https://www.who.int/emergencies/mers-cov/en/. Accessed 4 February 2020). The MERS case-fatality rate of 34.4% is about triple that of SARS, and persons in the 50–59 year age group are at highest risk for primary cases. In the short time from its emergence in December 2019 to 15 March 2020, the SARS-CoV-2 has been reported in 134 countries. At the time of writing, the situation was evolving rapidly, with over 142,000 confirmed cases reported globally (over 81,000 in China) and 3194 deaths in China (3.9% case-fatality rate) and over 2100 deaths outside of China. Of countries and continents outside of China, South Korea, Iran, and Europe (particularly Italy) have experienced a high number of COVID-19 cases (https://www.who.int/emergencies/diseases/novel-coronavirus-2019/situation-reports/. Accessed 15 March 2020). Mortality rates vary widely, and depend on the age of patients, underlying risk factors, and the denominator definition – hospitalized cases, all symptomatic cases, only moderate to severe cases, etc. In a study of adult patients (mean age 59.7 y; 40% with chronic illnesses) with SARS-CoV-2 pneumonia admitted to the intensive care unit (ICU), 61.5% died within 28 days [18]. In contrast, a study of hospitalized patients (median age 47.5 years) across Beijing showed 18% of cases to be severe and 73% mild, with a fatality rate of 0.9% [19]. Mortality is highest in older persons, with a median age of 59–75 years [15,17]. Treatment for all severe HCoV infections is supportive although a randomized, double-blinded, control clinical trial has been conducted on a Gilead drug Remdesivir [20] Based on one study focused on children, a total of 28 children aged from 1 month to 17 years have been reported in China. All paediatric cases with laboratory-confirmed SARS-CoV-2 infection were mild cases with no deaths reported [21]. During the first 2 months of the current outbreak, COVID-19 spread rapidly throughout China and caused varying degrees of illness with a death rate of 1.3%. Patients often presented without fever, and many did not have abnormal radiologic findings [22].

The Chinese Centers for Disease Control and Prevention team analysed more than 72,000 patient records, of which 44,672 were laboratory-confirmed cases, 16,186 suspected cases, 10,567 clinically diagnosed cases, and 889 asymptomatic cases. Of the confirmed cases, about 14% of the illnesses were severe, which included pneumonia and shortness of breath, and about 5% have the critical disease, marked by respiratory failure, septic shock, and multi-organ failure. The overall case-fatality rate was 2.3%, and of 1023 deaths included in the study, the majority were in people age 60 and older or those with underlying medical conditions http://www.cidrap.umn.edu/news-perspective/2020/02/more-outbreak-details-emerge-covid-19-cases-top-70000 (Accessed 18 February 2020).

## Laboratory diagnosis

### Specimen collection

It must be appreciated that no matter how accurate and fast laboratory testing methods are, the diagnosis of viral pneumonias such as caused by SARS-CoV-2 involves collecting the correct specimen from the patient at the right time. The endemic HCoVs have been detected from a variety of upper and lower respiratory sources including throat, nasal nasopharyngeal (NP), sputum, and bronchial fluid [12,23,24]. Wang et al have just reported that oropharyngeal (OP) swabs (*n* = 398) were used much more frequently than NP swabs (*n* = 8) in China during the COVID-19 outbreak; however, the SARS-CoV-2 RNA was detected only in 32% of OP swabs, which was significantly lower than that in NP swabs (63%)[25]. The US centers for disease control and prevention (CDC) recommends collecting the upper respiratory NP swab. Collection of an OP specimen is a lower priority, and, if collected, should be combined in the same tube as the NP swab (https://www.cdc.gov/coronavirus/2019-nCoV/lab/guidelines-clinical-specimens.html. Accessed 16 March 2020). Swab specimens should be placed in a universal or viral transport medium. Nasopharyngeal aspirates are also suitable specimens for the detection of HCoVs.

For the most sensitive detection of SARS-CoV, MERS-CoV, and SARS-CoV-2, the collection and testing of both upper and lower respiratory samples [sputum, bronchoalveolar lavage fluid (BAL)] is recommended [26]. However, the collection of sputum and particularly BAL via bronchoscopy increases biosafety risk to healthcare workers through the creation of aerosol droplets. Proper use of personal protective equipment (PPE) by healthcare workers is important. Bronchoscopy is a highly technical procedure requiring well-trained staff and may not be available in many parts of the world. Upper respiratory specimens are easy to collect, thereby increasing access to testing for patients with mild symptoms, and in the resource limited settings. SARS-CoV and MERS-CoV RNA are also detected from stool, urine and blood specimens, although generally less reliably than from respiratory specimens [26–28]. An exception is SARS-CoV RNA which is consistently detected in feces at about two weeks after symptom onset [26,29]. For the most sensitive detection of endemic HCoVs, upper respiratory specimens should be collected within the first few days of symptom onset. The dynamics of RNA shedding in MERS and SARS patients may reflect the specimen source, severity of illness, as well as underlying risk factors. Among hospitalized patients who did not require ventilator support, MERS-CoV RNA levels in the upper respiratory tract usually peaked in the first week after symptom onset. Among eventual fatal cases requiring ventilation, RNA levels in lower respiratory tract specimens peaked between weeks 2 and 3 [27]. Similar shedding patterns were seen for SARS-CoV: RNA positive rates peaked in upper respiratory tract specimens at 7–10 days after symptom onset and then steadily declined after that, while RNA positive rates in lower respiratory tract specimens remained higher throughout 3 weeks after onset of illness [26]. In one study, diabetes was associated with prolonged MERS-CoV RNA shedding in the respiratory tract [27].

Viral pneumonias typically do not result in the production of purulent sputum. Thus, a nasopharyngeal swab/wash is usually the collection method used to obtain a specimen for testing. Nasopharyngeal specimens may miss early infection; a deeper specimen may need to be obtained by bronchoscopy. Alternatively, repeated testing can be used because over time, the likelihood of the SARS-CoV-2 being present in the nasopharynx increases. Self-collected saliva specimens were tested positive in 11 of 12 COVID-19 patients, suggesting it is a promising non-invasive specimen for diagnosis, monitoring, and infection control in SARS-CoV-2 infections [30]. At the time of writing there was little data on the performance of upper vs. lower respiratory tract specimens for the detection of SARS-CoV-2 [16]. Serum is another source for the detection of SARS-CoV-2. However, only 15% of patients hospitalized with pneumonia had detectable RNA in serum [16]. Specimens collected for laboratory testing of HCoVs should be maintained at refrigerated temperature for up to 72 h, or frozen at −70°C or below (https://www.cdc.gov/coronavirus/2019-nCoV/lab/guidelines-clinical-specimens.html. Accessed 15 March 2020). Rectal specimens have been reported positive in patients infected with SARS-CoV-2 [20].

### Biosafety considerations

If the patient’s travel or exposure history or symptoms suggest possible infection with a high-risk, novel agent, SARS-CoV, or MERS-CoV, then the initial handling of the specimen should be performed under biosafety level 3 (BSL-3) conditions until the specimen or an aliquot is rendered noninfectious by lysis or another method. Virus isolation should not be routinely performed in this situation (https://www.asm.org/Articles/Policy/Laboratory-Response-Network-LRN-Sentinel-Level-C. Accessed 4 February 2020). The U.S. CDC biosafety guidelines state that routine diagnostic testing of specimens from suspected or confirmed SARS-CoV-2 patients, can be handled in a BSL-2 laboratory using standard precautions (https://www.cdc.gov/coronavirus/2019-nCoV/lab/lab-biosafety-guidelines.html. Accessed 21 March 2020).

### Cell culture

Isolation of HCoVs in cell culture is not routinely performed for diagnostic purposes due to the lack of permissive cell lines, time to results, labour and expertise requirements, and the lack of commercial antisera for culture confirmation (Table 2). SARS-CoV and MERS-CoV and SARS-CoV-2 will grow in primary monkey cells and cell lines such as Vero and LLC-MK2, but cell culture should not be performed for suspect cases in routine diagnostic laboratories for biosafety reasons [2,6,31,32]. However, virus isolation in cell cultures is critical to obtain isolates for characterization and to support the development of vaccines and therapeutic agents.

**Table 2.** Laboratory techniques for detection of coronaviruses.

Method	Characteristics	Test time	Application	Reference
Antigen EIA	Rapid, poor sensitivity, some are CLIA-waived	<30 min	Diagnosis (detection)	[33–35]
Antigen IFA	Good sensitivity and specificity, subjective interpretation	1–4 h	Diagnosis (detection)	[36,37]
Cell culture	Gold standard, pure culture for further research and development, time consuming	1–7 days	Diagnosis (detection, differentiation, typing and characterization) and research	[2,6,31,32]
Serology	Retrospective, cross-reaction	2–8 h	Infection confirmation, epidemiology and research, vaccine evaluation	[2,3,40–42]
NAAT, monoplex, pan-HCoV	High sensitivity with universal coverage of all species of HCoV	1–8 h	Diagnosis (detection), discovery and research	[52–54]
NAAT, monoplex, specific-HCoV	High sensitivity and specificity for special species, potential quantification	1–8 h	Diagnosis (detection, differentiation, and limited typing) and research	[69,70]
NAAT, multiplex	High sensitivity and specificity, covering other pathogens, FilmArray RP EZ is CLIA-waived	1–8 h	Diagnosis (detection, differentiation, and limited typing) and research	[12,55–57]
NAAT, POCT	Rapid and safe, good sensitivity and specificity, some are CLIA-waived	15–30 min	Diagnosis (detection and limited differentiation) and research	[63,67]

*Note:* EIA, enzyme immunoassay; IFA, immunofluorescent assay; NAAT, nucleic acid amplification test; CLIA, Clinical Laboratory Improvement Act.

### Rapid antigen tests

Rapid antigen tests would theoretically provide the advantage of fast time to results and low-cost detection of HCoVs but are likely to suffer from poor sensitivity based on the experience with this method for influenza (Flu) viruses [33–37] (Table 2). In a pre-peer reviewed article, Diao et al. reported that a fluorescence immunochromatographic assay is an accurate, rapid, early and simple method for detecting nucleocapsid protein of SARS-CoV-2 in NP swab for diagnosis of COVID-19 (https://www.medrxiv.org/content/10.1101/2020.03.07.20032524v2. Accessed 15 March 2020). The incorporation of colloidal gold-labeled immunoglobulin G (IgG) as the detection reagent is an approach that may increase the sensitivity of rapid antigen tests for respiratory viruses [38]. Monoclonal antibodies specifically against SARS-CoV-2 have been under preparation. Novel approaches to concentrate antigen, or to amplify the detection phase are needed if these methods are to have clinical utility. Sona Nanotech (Halifax, Canada) is developing a quick-response lateral-flow test to screen COVID-19 patients targeting to produce results in 5–15 min (https://sonanano.com/sona-develops-rapid-screening-test-for-coronavirus/. Accessed 15 February 2020). Timing of specimen collection, when viral titres are highest, may improve the diagnostic sensitivity of rapid antigen tests for HCoVs [39].

### Serology

Serological assays are not routinely used for diagnosis of HCoV infections due to the lack of commercial reagents, let alone commercial reagents that have been vetted by clinical trials and the regulatory review process [40,41] (Table 2). Serological assays, on the other hand, are important for understanding the epidemiology of emerging HCoVs, including the burden and role of asymptomatic infections.

It has been particularly important for antibody detection in the diagnosis of cases of novel and emerging HCoVs, such as SARS-CoV and MERS-CoV [2,3]. In these situations, affected patients may not test positive for viral RNA, particularly in the early phase of the disease, but retrospectively can be shown to have developed an immune response. When SARS-CoV-2 was identified, especially when rapid antigen testing and/or molecular assays are neither available nor stable, serology can be used as a supplementary diagnostic tool. A recent study demonstrated that both IgM and IgG antibodies were detected 5 days after onset in all 39 patients infected with SARS-CoV-2 infection. The authors recommended to use serology to facilitate the diagnosis of SARS-CoV-2 infections when an NP swab specimen was collected inappropriately and the molecular assays were performed unsatisfactorily [42]. In China, six serology devices have just received urgent approval from the National Medical Products Administration (NMPA) by 12 March 2020 (Table 3). Proper specimen handling and storage are important to maintain the integrity of specimens and the performance of serologic tests.

**Table 3.** Diagnostic devices cleared in China for laboratory diagnosis of SARS-CoV-2 infections.

Registration number	Manufacturer	Date registered	Specimen type	Principle and method	Instrument	Targets	Remarks
20203400057	Shanghai ZJ Bio-Tech	26 January 2020	Sputum, BAL, NPS	Fluorescence RT-PCR	Real-time thermocycler, e.g. ABI 7500 Fast Dx Real-Time PCR Instrument	ORF1ab, E, N	LOD: 1000 copies/ml
20203400058	Shanghai GeneoDx Biotech	26 January 2020	Sputum, pharyngeal swab	Fluorescence RT-PCR	Real-time thermocycler, e.g. ABI 7500 Fast Dx Real-Time PCR Instrument	ORF1ab, N	
20203400060	BGI Biotech (Wuhan)	26 January 2020	BAL, pharyngeal swab	Fluorescence RT-PCR	Real-time thermocycler, e.g. ABI 7500 Fast Dx Real-Time PCR Instrument	ORF1ab	Single target
20203400061	MGI Tech	26 January 2020	Undefined	NGS	Genetic sequencer (DNBSEQ-T7)	Microbial DNA and RNA including SARS-CoV-2 genome	
20203400063	Da An Gene	28 January 2020	Pharyngeal swab, sputum	Fluorescence RT-PCR	Real-time thermocycler, e.g. ABI 7500 Fast Dx Real-Time PCR Instrument	ORF1ab, N and IC	LOD, 500 copies/ml
20203400064	Sansure Biotech	28 January 2020	NPS, BAL	Fluorescence RT-PCR	Real-time thermocycler, e.g. ABI 7500 Fast Dx Real-Time PCR Instrument	ORF1ab, N, IC	LOD, 200 copies/ml; One-step RNA with 10 min specimen pretreatment
20203400065	Shanghai BioGerm Medical Biotech	31 January 2020	NPS, OPS, sputum	Fluorescence RT-PCR	Real-time thermocycler, e.g. ABI 7500 Fast Dx Real-Time PCR Instrument	ORF1ab, N	
20203400176	Wongfo Biotech	22 February 2020	Serum, plasma, whole blood	Immune colloidal gold technique	Not needed	Antibody against SARS-CoV-2	
20203400177	Innovita Biological Technology	22 February 2020	Serum, plasma	Immune colloidal gold technique	Not needed	IgM/IgG antibody against SARS-CoV-2	
20203400178	CapitalBio (Chengdu)	22 February 2020	NPS	Isothermal amplification and microarray	RTisochip™-A (20173401354)	S, N and IC. Also covers Flu A (universal, H1N1, H3N2), Flu B and RSV	LOD, 50 copies/reaction; Total TAT,1.5 h
20203400179	Beijing Applied Biological Technologies (X-ABT)	27 February 2020	Sputum, NPS	Fluorescence RT-PCR	Real-time thermocycler, e.g. ABI 7500 Fast Dx Real-Time PCR Instrument	ORF1ab, N, E, IC	LOD, 200 copies/ml; TAT, 90 min
20203400182	Bioscience (Chongqing)	1 March 2020	Serum	Magnetic particle chemiluminescence	Automated magnetic analyser: Axceed 260	IgM antibody against SARS-CoV-2	
20203400183	Bioscience (Chongqing)	1 March 2020	Serum	Magnetic particle chemiluminescence	Automated magnetic analyser: Axceed 260	IgG antibody against SARS-CoV-2	
20203400184	Maccura Biotechnology	1 March 2020	Pharyngeal swab, sputum	Fluorescence RT-PCR	Real-time thermocycler, e.g. ABI 7500 Fast Dx Real-Time PCR Instrument	ORF1ab, N, E	
20203400198	Xiamen Wantai Kairui Biotechnology	6 March 2020	Serum, plasma	Chemiluminescence immunoassay	Caris 200 Automatic Chemiluminescence Analyser	Total antibody (IgM, IgG and IgA) against SARS-CoV-2	TAT, 29 min; Throughput, 400 tests/hour; Sensitivity, 94.8%; Specificity, 99.7%
20203400199	Guangdong Hecin-Scientific	11 March 2020	Serum, plasma	Immune colloidal gold technique	Not needed	IgM antibody against SARS-CoV-2	
20203400212	Wuhan Easydiagnosis Biomedicine	12 March 2020	NPS, OPS, sputum	Fluorescence RT-PCR	Real-time thermocycler, e.g. ABI 7500 Fast Dx Real-Time PCR Instrument	ORF1ab, N	

*Note:* LOD, limit of detection; TAT, turnaround time; NPS, nasopharyngeal swabs; OPS, oropharyngeal swabs; BAL, bronchoalveolar lavage; NGS, next-generation sequencing; Flu, influenza; RSV, respiratory syncytial virus.

### Molecular methods

Random-amplification deep-sequencing approaches played a critical role in identifying MERS-CoV and SARS-CoV-2 [6,11,43–47]. For the clinical diagnostic application, the genetic heterogeneity of HCoVs precludes a single “pan-HCoV” molecular assay [48–51] (Table 2). Some pan-CoV assays use degenerate primers [52], some utilize multiple primer sets [53], and others employ a single set of nondegenerate primers [54]. Current molecular respiratory panels that detect the endemic HCoVs (HCoV-NL63, HCoV-HKU1, HCoV-OC43, and HCoV-229E) require multiple sets of PCR oligonucleotides [12,55–57]. SARS-CoV-2 cases tested negative for endemic HCoVs included in molecular respiratory panels [10].

In China, at the time of revising, eleven molecular devices from Shanghai ZJ Bio-Tech, Shanghai GeneoDx Biotech, BGI Biotech (Wuhan), MGI Tech, Da An Gene, Sansure Biotech, Shanghai BioGerm Medical Biotech Capitalbio (Chengdu), Beijing Applied Biological Technologies, Maccura Biotechnology, and Wuhan Easydiagnosis Biomedicine have received urgent approval from NMPA and their characteristics are contrasted in Table 3. Variable performance has been reported on these devices [47,58]. In their registration certificates, it was clearly indicated that the certificate was for urgent and supplemental diagnosis of pneumonia caused by SARS-CoV-2. Additional multi-centre clinical trial data are needed for extension after one year. Among them, one (MGI Tech) uses its NGS technique to detect all pathogens in a given specimen including SARS-CoV-2 and the other one (Innovita) uses its isothermal amplification followed by chip detection. The other nine devices incorporated real-time PCR technique with hydrolysis probes. After nucleic acids get extracted (separated reagents and systems), the extracts are transferred to a real-time PCR thermocycler (e.g. ABI 7500 Fast Dx Real-Time PCR Instrument) for nucleic acid amplification and detection.

Several RT-PCR protocols for detection of SARS-CoV-2 RNA have been posted by the World Health Organization at https://www.who.int/emergencies/diseases/novel-coronavirus-2019/technical-guidance/laboratory-guidance. (Accessed 15 March 2020). Three of these protocols are listed below.

The US CDC developed developed a RT-PCR Diagnostic Panel for universal detection of SARS-like betacoronaviruses and specific detection of SARS-CoV-2 [20]. Three separate RT-PCR reactions target the N gene. One primer/probe set detects all betacoronaviruses, while two sets are specific for SARS-CoV-2. All 3 assays must be positive to report presumptive positive for SARS-CoV-2 (https://www.fda.gov/media/134922/download. Accessed 15 March 2020). Specimen types included upper and lower respiratory specimens (such as NP or OP swabs, sputum, lower respiratory tract aspirates, BAL, and nasopharyngeal wash/aspirate or nasal aspirate). It received emergency use authorization (EUA) on 4 February 2020. At the time of revision, the US Food and Drug Administration (FDA) has granted thirteen in vitro diagnostics EUAs: the aforementioned CDC assay; the New York SARS-CoV-2 Real-time RT-PCR Diagnostic Panel (Wadsworth Center, New York State Department of Health); TaqPath COVID-19 Combo Kit (Thermo Fisher Scientific, Inc.); cobas SARS-CoV-2 (Roche Molecular Systems, Inc.; Panther Fusion SARS-CoV-2 (Hologic, Inc.); COVID-19 RT-PCR Test (Laboratory Corporation of America); Lyra SARS-CoV-2 Assay (Quidel Corp.); Quest SARS-CoV-2 rRT-PCR (Quest Diagnostics Infectious Disease, Inc.); Abbott RealTime SARS-CoV-2 assay (Abbott Molecular); Simplexa COVID-19 Direct (DiaSorin Molecular LLC); ePlex SARS-CoV-2 Test (GenMark Diagnostics, Inc.); Primerdesign Ltd COVID-19 genesig Real-Time PCR assay (Primerdesign Ltd); and Xpert Xpress SARS-CoV-2 test (Cepheid) (https://www.fda.gov/medical-devices/emergency-situations-medical-devices/emergency-use-authorizations#coronavirus2019. Accessed 21 March 2020). Additional in vitro diagnostic assays are in development.

The Charité algorithm (Berlin, Germany) begins with two RT-PCR assays that detect E and RdRp genes of subgenus *Sarbecovirus* (SARS-CoV, SARS-CoV-2, and bat-associated betacoronaviruses). Both assays must be positive to advance to the next step in the testing algorithm. The second step consists of a SARS-CoV-2 specific RT-PCR that targets RdRp [59,60]. Exclusivity testing showed that alphacoronaviruses (CoV-NL63 and −229E) and betacoronaviruses HCoV-OC43, HCoV-HKU1 and MERS-CoV were not detected (https://www.who.int/docs/default-source/coronaviruse/protocol-v2-1.pdf?sfvrsn=a9ef618c_2. Accessed 8 February 2020).

*The University of Hong Kong Li Ka Shing Faculty of Medicine* protocol uses two assays (N gene screening assay followed by Orf1b assay for confirmation) to detect subgenus *Sarbecovirus* [30,61]. Since SARS-CoV is not circulating in humans currently, cases that are positive should be considered as SARS-CoV-2 infected cases. Exclusivity testing showed that 229E, OC43 and MERS, 229E, HKU1, NL63, OC43 yielded negative results (https://www.who.int/docs/default-source/coronaviruse/peiris-protocol-16-1-20.pdf?sfvrsn=af1aac73_4. Accessed 8 February 2020).

## Future direction

**Figure 1.**
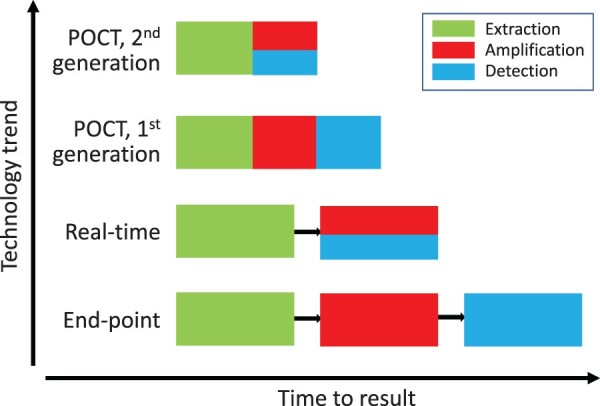
Evolutions in molecular testing procedures. The point-of-care test (POCT) devices incorporate nucleic acid extraction, amplification and detection together into an integrated and sealed cartridge making it simple, rapid and safe. During end-point PCR, DNA is detected or measured at the completion of PCR amplification, requiring post-PCR processing. Real-time PCR is a closed-tube system in which DNA is detected or measured during the exponential phase of amplification.

All three novel coronaviruses are highly contagious. Fast, safe, simple to use diagnostic devices performed at or near the point of care (POC) (Figure 1) which have been shown to impact patient management and control of infectious disease epidemics [62], are extremely desirable in POC when biosafety facility is limited (Table 3). Several manufactures have been spending efforts to generate devices for POC testing (POCT) [63]. The ID NOW™ (previously Alere i) Influenza A & B assay (Abbott, San Diego, CA) was cleared by the US FDA for direct use on NP swabs as the first-ever Clinical Laboratory Improvement Amendments (CLIA)-waived nucleic acid-based test in January 2016 [64,65]. Similarly, the Xpert^®^ Xpress Flu/RSV (Cepheid, Sunnyvale, CA) and cobas^®^ Liat^®^ Flu A/B & RSV (Roche Molecular Systems, Pleasanton, CA) assays are integrated nucleic acid extraction-independent devices that have recently received FDA clearance and CLIA-waiver for simultaneous detection and identification of FluA, FluB, and RSV in nasopharyngeal swabs [66]. The FilmArray^®^ Respiratory EZ Panel (BioFire, Salt Lake City, UT) so far so far is the only CLIA-waived syndromic panel that covers a set of 14 respiratory viral and bacterial pathogens including classical coronavirus species [67].

Considering the increased levels of mortality and infectivity associated with three novel-coronavirus outbreaks, these random-access, safe and simple tests, which offer fast and accurate detection and identification, are likely to have an immediate impact on prompt clinical and epidemiological decisions [7,63]. Lysis buffer can be used to inactivate the infectivity of specimens so the testing can be run at POC when a biosafety cabinet is not available. Fast near-patient and POCT could help more efficiently triage of suspected cases of novel coronavirus, helping to focus limited resources on enabling appropriate use of quarantine. A handful of diagnostics developers are now striving to bring fast SARS-CoV-2 tests to market as soon as possible, with hopes of ultimately assisting with the ongoing outbreak in China. Molecular diagnostic tests for use at the are in development from Cepheid and HiberGene (Dublin, Ireland). Cepheid has some advantages in the molecular POCT space because it already has instruments placed in China. Mobidiag, meanwhile, may offer additional benefits with a multiplex test for coronavirus and Flu viruses (https://www.genomeweb.com/pcr/diagnostics-firms-rush-develop-rapid-point-care-tests-novel-coronavirus#.XkeA3SgzY2x. Accessed 15 February 2020).

## References

[1] Su S, Wong G, Shi W, et al. Epidemiology, genetic recombination, and Pathogenesis of coronaviruses. Trends Microbiol 2016;24(6):490–502. doi:10.1016/j.tim.2016.1003.1003 doi: 10.1016/j.tim.2016.1003.1003 27012512 PMC7125511

[2] Ksiazek TG, Erdman D, Goldsmith CS, et al. A novel coronavirus associated with severe acute respiratory syndrome. N Engl J Med. 2003;348(20):1953–1966. doi:10.1056/NEJMoa030781. 12690092

[3] Peiris JS, Lai ST, Poon LL, et al. Coronavirus as a possible cause of severe acute respiratory syndrome. Lancet. 2003;361(9366):1319–1325. doi: 10.1016/S0140-6736(03)13077-2 12711465 PMC7112372

[4] Assiri A, Al-Tawfiq JA, Al-Rabeeah AA, et al. Epidemiological, demographic, and clinical characteristics of 47 cases of Middle East respiratory syndrome coronavirus disease from Saudi Arabia: a descriptive study. Lancet Infect Dis. 2013;13(9):752–761. doi: 10.1016/S1473-3099(13)70204-4 23891402 PMC7185445

[5] Drosten C, Seilmaier M, Corman VM, et al. Clinical features and virological analysis of a case of Middle East respiratory syndrome coronavirus infection. Lancet Infect Dis. 2013;13(9):745–751. doi: 10.1016/S1473-3099(13)70154-3 23782859 PMC7164791

[6] Zaki AM, van Boheemen S, Bestebroer TM, et al. Isolation of a novel coronavirus from a man with pneumonia in Saudi Arabia. N Engl J Med. 2012;367(19):1814–1820. doi:10.1056/NEJMoa1211721. 23075143

[7] Lu H, Stratton CW, Tang YW. Outbreak of pneumonia of unknown Etiology in Wuhan China: the Mystery and the Miracle. J Med Virol. 2020;92(4):401–402. doi: 10.1002/jmv.25678 31950516 PMC7166628

[8] Zhu N, Zhang D, Wang W, et al. A novel coronavirus from patients with pneumonia in China, 2019. N Engl J Med. 2020;382(8):727–733. doi: 10.1056/NEJMoa2001017 31978945 PMC7092803

[9] Anonymous. The species severe acute respiratory syndrome-related coronavirus: classifying 2019-nCoV and naming it SARS-CoV-2. Nat Microbiol. 2020. Epub ahead of print. 10.1038/s41564-020-0695-z PMC7095448 32123347

[10] Lu R, Zhao X, Li J, et al. Genomic characterisation and epidemiology of 2019 novel coronavirus: implications for virus origins and receptor binding. Lancet. 2020;30(20):30251–30258. 10.1016/S0140-6736(20)30251-8 PMC7159086 32007145

[11] Zhou P, Yang XL, Wang XG, et al. A pneumonia outbreak associated with a new coronavirus of probable bat origin. Nature. 2020;579(7798):270–273. doi: 10.1038/s41586-020-2012-7 32015507 PMC7095418

[12] Gaunt ER, Hardie A, Claas EC, et al. Epidemiology and clinical presentations of the four human coronaviruses 229E, HKU1, NL63, and OC43 detected over 3 years using a novel multiplex real-time PCR method. J Clin Microbiol. 2010;48(8):2940–2947. doi: 10.1128/JCM.00636-10 20554810 PMC2916580

[13] Wong G, Liu W, Liu Y, et al. MERS, SARS, and Ebola: The role of super-spreaders in infectious disease. Cell Host Microbe. 2015;18(4):398–401. doi: 10.1016/j.chom.2015.09.013 26468744 PMC7128246

[14] Sun J, He W, Wang L, et al. COVID-19: epidemiology, evolution, and cross-disciplinary perspectives. Trends Mol Med. 2020. Epub ahead of print. 10.1016/j.molmed.2020.02.008 PMC7118693 32359479

[15] Li Q, Guan X, Wu P, et al. Early transmission dynamics in Wuhan, China, of novel coronavirus-infected pneumonia. N Engl J Med. 2020. Epub ahead of print. 10.1056/NEJMoa2001316 PMC7121484 31995857

[16] Huang C, Wang Y, Li X, et al. Clinical features of patients infected with 2019 novel coronavirus in Wuhan, China. Lancet. 2020;24(20):30183–30185. 10.1016/S0140-6736(20)30183-5 PMC7159299 31986264

[17] Wang W, Tang J, Wei F. Updated understanding of the outbreak of 2019 novel coronavirus (2019-nCoV) in Wuhan, China. J Med Virol. 2020;92(4):441–447. doi: 10.1002/jmv.25689 31994742 PMC7167192

[18] Yang X, Yu Y, Xu J, et al. Clinical course and outcomes of critically ill patients with SARS-CoV-2 pneumonia in Wuhan, China: a single-centered, retrospective, observational study. Lancet Respir Med. 2020. Epub ahead of print. 10.1016/S2213-2600(20)30079-5 PMC7102538 32105632

[19] Tian S, Hu N, Lou J, et al. Characteristics of COVID-19 infection in Beijing. J Infect. 2020;27(20):30101–30108. 10.1016/j.jinf.2020.02.018 PMC7102527 32112886

[20] Holshue ML, DeBolt C, Lindquist S, et al. First case of 2019 novel coronavirus in the United States. N Engl J Med. 2020;382(10):929–936. doi: 10.1056/NEJMoa2001191 32004427 PMC7092802

[21] Shen K, Yang Y, Wang T, et al. Diagnosis, treatment, and prevention of 2019 novel coronavirus infection in children: experts’ consensus statement. World J Pediatr. 2020. Epub ahead of print. 10.1007/s12519-020-00343-7 PMC7090771 32034659

[22] Guan WJ, Ni ZY, Hu Y, et al. Clinical characteristics of coronavirus disease 2019 in China. N Engl J Med. 2020. Epub ahead of print. 10.1056/NEJMoa2002032 PMC7092819 32109013

[23] Charlton CL, Babady E, Ginocchio CC, et al. Practical guidance for clinical microbiology laboratories: viruses causing acute respiratory tract infections. Clin Microbiol Rev. 2019;32(1). doi: 10.1128/CMR.00042-18 PMC6302358 30541871

[24] Falsey AR, Formica MA, Walsh EE. Simple method for combining sputum and nasal samples for virus detection by reverse transcriptase PCR. J Clin Microbiol. 2012;50(8):2835. doi: 10.1128/JCM.01473-12 22692748 PMC3421505

[25] Wang W, Xu Y, Gao R, et al. Detection of SARS-CoV-2 in different types of clinical specimens. Jama. 2020. Epub ahead of print. 10.1001/jama.2020.3786 PMC7066521 32159775

[26] Cheng PK, Wong DA, Tong LK, et al. Viral shedding patterns of coronavirus in patients with probable severe acute respiratory syndrome. Lancet. 2004;363(9422):1699–1700. doi: 10.1016/S0140-6736(04)16255-7 15158632 PMC7112423

[27] Al-Abdely HM, Midgley CM, Alkhamis AM, et al. Middle East respiratory syndrome coronavirus infection dynamics and antibody Responses among clinically Diverse patients, Saudi Arabia. Emerg Infect Dis. 2019;25(4):753–766. doi:10.3201/eid2504.181595. 30882305 PMC6433025

[28] Poissy J, Goffard A, Parmentier-Decrucq E, et al. Kinetics and pattern of viral excretion in biological specimens of two MERS-CoV cases. J Clin Virol. 2014;61(2):275–278. doi: 10.1016/j.jcv.2014.07.002 25073585 PMC7106432

[29] Xu D, Zhang Z, Jin L, et al. Persistent shedding of viable SARS-CoV in urine and stool of SARS patients during the convalescent phase. Eur J Clin Microbiol Infect Dis. 2005;24(3):165–171. doi:10.1007/s10096-10005-11299-10095 doi: 10.1007/s10096-10005-11299-10095 15789222 PMC7088045

[30] To KK, Tsang OT, Chik-Yan Yip C, et al. Consistent detection of 2019 novel coronavirus in saliva. Clin Infect Dis. 2020. Epub ahead of print. 10.1093/cid/ciaa149 PMC7108139 32047895

[31] Hamre D, Kindig DA, Mann J. Growth and intracellular development of a new respiratory virus. J Virol. 1967;1(4):810–816. doi: 10.1128/JVI.1.4.810-816.1967 4912236 PMC375356

[32] Tyrrell DAJ, Bynoe ML. Cultivation of a novel type of common-cold virus in organ cultures. Br Med J. 1965;1(5448):1467–1470. doi: 10.1136/bmj.1.5448.1467 14288084 PMC2166670

[33] Chen Y, Chan KH, Hong C, et al. A highly specific rapid antigen detection assay for on-site diagnosis of MERS. J Infect. 2016;73(1):82–84. doi:10.1016/j.jinf.2016.1004.1014 doi: 10.1016/j.jinf.2016.1004.1014 27144915 PMC7127149

[34] Lau SK, Woo PC, Wong BH, et al. Detection of severe acute respiratory syndrome (SARS) coronavirus nucleocapsid protein in SARS patients by enzyme-linked immunosorbent assay. J Clin Microbiol. 2004;42(7):2884–2889. doi: 10.1128/JCM.42.7.2884-2889.2004 15243033 PMC446266

[35] Sastre P, Dijkman R, Camunas A, et al. Differentiation between human coronaviruses NL63 and 229E using a novel double-antibody sandwich enzyme-linked immunosorbent assay based on specific monoclonal antibodies. Clin Vaccine Immunol. 2011;18(1):113–118. doi: 10.1128/CVI.00355-10 21084464 PMC3019789

[36] Liu IJ, Chen PJ, Yeh SH, et al. Immunofluorescence assay for detection of the nucleocapsid antigen of the severe acute respiratory syndrome (SARS)-associated coronavirus in cells derived from throat wash samples of patients with SARS. J Clin Microbiol. 2005;43(5):2444–2448. doi: 10.1128/JCM.43.5.2444-2448.2005 15872279 PMC1153760

[37] Sizun J, Arbour N, Talbot PJ. Comparison of immunofluorescence with monoclonal antibodies and RT-PCR for the detection of human coronaviruses 229E and OC43 in cell culture. J Virol Methods. 1998;72(2):145–152. doi: 10.1016/S0166-0934(98)00013-5 9694322 PMC7119642

[38] Li W, Liu L, Chen L, et al. Evaluation of a commercial colloidal gold assay for detection of influenza A and B virus in children's respiratory specimens. Fetal Pediatr Pathol. 2019;15:1–6. doi: 10.1080/15513815.2019.1639088 31304835

[39] Bruning AHL, Aatola H, Toivola H, et al. Rapid detection and monitoring of human coronavirus infections. New Microbes New Infect. 2018;24:52–55. doi:10.1016/j.nmni.2018.1004.1007 doi: 10.1016/j.nmni.2018.1004.1007 29872531 PMC5986163

[40] Chan CM, Tse H, Wong SS, et al. Examination of seroprevalence of coronavirus HKU1 infection with S protein-based ELISA and neutralization assay against viral spike pseudotyped virus. J Clin Virol. 2009;45(1):54–60. doi:10.1016/j.jcv.2009.1002.1011 doi: 10.1016/j.jcv.2009.1002.1011 19342289 PMC7108224

[41] Shao X, Guo X, Esper F, et al. Seroepidemiology of group I human coronaviruses in children. J Clin Virol. 2007;40(3):207–213. doi: 10.1016/j.jcv.2007.08.007 17889596 PMC2100388

[42] Zhang W, Du RH, Li B, et al. Molecular and serological investigation of 2019-nCoV infected patients: implication of multiple shedding routes. Emerg Microbes Infect. 2020;9(1):386–389. doi: 10.1080/22221751.2020.1729071 32065057 PMC7048229

[43] Briese T, Mishra N, Jain K, et al. Middle East respiratory syndrome coronavirus quasispecies that include homologues of human isolates revealed through whole-genome analysis and virus cultured from dromedary camels in Saudi Arabia. mBio. 2014;5(3):e01146–e01114. doi: 10.1128/mBio.01146-14 24781747 PMC4010836

[44] Chen L, Liu W, Zhang Q, et al. RNA based mNGS approach identifies a novel human coronavirus from two individual pneumonia cases in 2019 Wuhan outbreak. Emerg Microbes Infect. 2020;9(1):313–319. doi: 10.1080/22221751.2020.1725399 32020836 PMC7033720

[45] Wu F, Zhao S, Yu B, et al. A new coronavirus associated with human respiratory disease in China. Nature. 2020;579(7798):265–269. doi: 10.1038/s41586-020-2008-3 32015508 PMC7094943

[46] Ren LL, Wang YM, Wu ZQ, et al. Identification of a novel coronavirus causing severe pneumonia in human: a descriptive study. Chin Med J. 2020. Epub ahead of print. 10.1097/CM9.0000000000000722 PMC7147275 32004165

[47] Wu X, Cai Y, Huang X, et al. Co-infection with SARS-CoV-2 and influenza A virus in patient with pneumonia, China. Emerg Infect Dis. 2020;26(6). doi: 10.3201/eid2606.200299 PMC7258479 32160148

[48] Das S, Dunbar S, Tang YW. Laboratory diagnosis of respiratory tract infections in children - the State of the Art. Front Microbiol. 2018;9:2478. doi:10.3389/fmicb.2018.02478. 30405553 PMC6200861

[49] Mahony JB, Richardson S. Molecular diagnosis of severe acute respiratory syndrome: the state of the art. J Mol Diagn. 2005;7(5):551–559. doi: 10.1016/S1525-1578(10)60587-9 16258152 PMC1867551

[50] Wu W, Tang YW. Emerging molecular assays for detection and characterization of respiratory viruses. Clin Lab Med. 2009;29(4):673–693. doi: 10.1016/j.cll.2009.07.005 19892228 PMC7130760

[51] Yan Y, Zhang S, Tang YW. Molecular assays for the detection and characterization of respiratory viruses. Semin Respir Crit Care Med. 2011;32(4):512–526. doi:10.1055/s-0031-1283288. 21858753

[52] Zlateva KT, Coenjaerts FE, Crusio KM, et al. No novel coronaviruses identified in a large collection of human nasopharyngeal specimens using family-wide CODEHOP-based primers. Arch Virol. 2013;158(1):251–255. doi:10.1007/s00705-00012-01487-00704 doi: 10.1007/s00705-00012-01487-00704 23053517 PMC7087030

[53] Kuypers J, Martin ET, Heugel J, et al. Clinical disease in children associated with newly described coronavirus subtypes. Pediatrics. 2007;119(1):e70–e76. doi:10.1542/peds.2006-1406. 17130280

[54] Woo PC, Lau SK, Chu CM, et al. Characterization and complete genome sequence of a novel coronavirus, coronavirus HKU1, from patients with pneumonia. J Virol. 2005;79(2):884–895. doi: 10.1128/JVI.79.2.884-895.2005 15613317 PMC538593

[55] Babady NE, England MR, Jurcic Smith KL, et al. Multicenter evaluation of the ePlex respiratory pathogen panel for the detection of viral and bacterial respiratory tract pathogens in nasopharyngeal swabs. J Clin Microbiol. 2018;56(2). doi: 10.1128/JCM.01658-17 PMC5786739 29212701

[56] Babady NE, Mead P, Stiles J, et al. Comparison of the Luminex xTAG RVP fast assay and the Idaho Technology FilmArray RP assay for detection of respiratory viruses in pediatric patients at a cancer hospital. J Clin Microbiol. 2012;50(7):2282–2288. doi: 10.1128/JCM.06186-11 22518855 PMC3405573

[57] Tang YW, Gonsalves S, Sun JY, et al. Clinical evaluation of the Luminex NxTAG respiratory pathogen panel. J Clin Microbiol. 2016;54(7):1912–1914. doi: 10.1128/JCM.00482-16 27122378 PMC4922127

[58] Liu R, Han H, Liu F, et al. Positive rate of RT-PCR detection of SARS-CoV-2 infection in 4880 cases from one hospital in Wuhan, China, from Jan to Feb 2020. Clin Chim Acta. 2020. Epub ahead of print. 10.1016/j.cca.2020.03.009 PMC7094385 32156607

[59] Rothe C, Schunk M, Sothmann P, et al. Transmission of 2019-nCoV infection from an asymptomatic contact in Germany. N Engl J Med. 2020;382(10):970–971. doi: 10.1056/NEJMc2001468 32003551 PMC7120970

[60] Corman VM, Landt O, Kaiser M, et al. Detection of 2019 novel coronavirus (2019-nCoV) by real-time RT-PCR. Euro Surveill. 2020;25(3). doi: 10.2807/1560-7917.ES.2020.25.3.2000045 PMC6988269 31992387

[61] Chan JF, Kok KH, Zhu Z, et al. Genomic characterization of the 2019 novel human-pathogenic coronavirus isolated from a patient with atypical pneumonia after visiting Wuhan. Emerg Microbes Infect. 2020;9(1):221–236. doi: 10.1080/22221751.2020.1719902 31987001 PMC7067204

[62] Raftery P, Condell O, Wasunna C, et al. Establishing Ebola virus disease (EVD) diagnostics using GeneXpert technology at a mobile laboratory in Liberia: impact on outbreak response, case management and laboratory systems strengthening. PLoS Negl Trop Dis. 2018;12(1):e0006135. doi: 10.1371/journal.pntd.0006135 29304039 PMC5755746

[63] Kozel TR, Burnham-Marusich AR. Point-of-care testing for infectious diseases: past, present, and future. J Clin Microbiol. 2017;55(8):2313–2320. doi: 10.1128/JCM.00476-17 28539345 PMC5527409

[64] Nie S, Roth RB, Stiles J, et al. Evaluation of Alere i influenza A&B for rapid detection of influenza viruses A and B. J Clin Microbiol. 2014;52(9):3339–3344. doi: 10.1128/JCM.01132-14 24989611 PMC4313160

[65] Wang H, Deng J, Tang YW. Profile of the Alere i influenza A & B assay: a pioneering molecular point-of-care test. Expert Rev Mol Diagn. 2018;18(5):403–409. doi: 10.1080/14737159.2018.1466703 29688086 PMC6153442

[66] Ling L, Kaplan SE, Lopez JC, et al. Parallel validation of three molecular devices for simultaneous detection and identification of influenza A and B and respiratory syncytial viruses. J Clin Microbiol. 2018;56(3). doi: 10.1128/JCM.01691-17 PMC5824037 29263204

[67] Beal SG, Posa M, Gaffar M, et al. Performance and impact of a CLIA-waived, point-of-care respiratory PCR Panel in a pediatric clinic. Pediatr Infect Dis J. 2020;39(3):188–191. doi:10.1097/INF.0000000000002544. 31929382

[68] van der Hoek L, Pyrc K, Jebbink MF, et al. Identification of a new human coronavirus. Nat Med. 2004;10(4):368–373. doi:10.1038/nm1024. 15034574 PMC7095789

[69] Chan JF, Choi GK, Tsang AK, et al. Development and evaluation of novel real-time reverse transcription-PCR assays with Locked nucleic acid probes targeting leader sequences of human-pathogenic coronaviruses. J Clin Microbiol. 2015;53(8):2722–2726. doi: 10.1128/JCM.01224-15 26019210 PMC4508434

[70] Dare RK, Fry AM, Chittaganpitch M, et al. Human coronavirus infections in rural Thailand: a comprehensive study using real-time reverse-transcription polymerase chain reaction assays. J Infect Dis. 2007;196(9):1321–1328. doi:10.1086/521308. 17922396 PMC7109921

